# Efficacy and Efficiency of Nutritional Support Teams

**DOI:** 10.3390/jcm8091281

**Published:** 2019-08-22

**Authors:** Emilie Reber, Rachel Strahm, Lia Bally, Philipp Schuetz, Zeno Stanga

**Affiliations:** 1 Department of Diabetes, Endocrinology, Nutritional Medicine and Metabolism, Bern University Hospital, and University of Bern, Freiburgstrasse 15, 3010 Bern, Switzerland; 2 Department of Medical University, Division of General Internal and Emergency Medicine, Kantonsspital Aarau, Tellstrasse 25, 5000 Aarau, Switzerland; 3 Department for Clinical Research, Medical Faculty, University of Basel, 4001 Basel, Switzerland

**Keywords:** nutritional support team, nutritional management, malnutrition, efficacy

## Abstract

Malnutrition is frequent in patients during a hospital admission and may further worsen during the hospital stay without appropriate nutritional support. Malnutrition causes greater complication rates, morbidity, and mortality rates, which increases the length of hospital stay and prolongs rehabilitation. Early recognition of individual nutritional risk and timely initiation of a tailored nutritional therapy are crucial. Recent evidence from large-scale trials suggests that efficient nutritional management not only improves the nutritional status, but also prevents negative clinical outcomes and increases patients’ quality of life. Multifaceted clinical knowledge is required to ensure optimal nutritional support, according to a patient’s individual situation and to avoid potential complications. Furthermore, clear definition of responsibilities and structuring of patient, and work processes are indispensable. Interdisciplinary and multiprofessional nutritional support teams have been built up to ensure and improve the quality and safety of nutritional treatments. These teams continuously check and optimize the quality of procedures in the core areas of nutritional management by implementing nutritional screening processes using a validated tool, nutritional status assessment, an adequate nutritional care plan development, prompt and targeted nutritional treatment delivery, and provision of accurate monitoring to oversee all aspects of care, from catering to artificial nutrition. The foundation of any nutritional care plan is the identification of patients at risk. The aim of this narrative review is to provide an overview about composition, tasks, and challenges of nutritional support teams, and to discuss the current evidence regarding their efficiency and efficacy in terms of clinical outcome and cost effectiveness.

## 1. Introduction

Malnutrition, which is defined as a state resulting from the lack of intake or uptake of nutrition leading to altered body composition, decreased mental and physical function, and impaired clinical outcome, is a lurking threat at hospitals in developing countries as well as in industrialized countries [1,2,3,4,5]. Up to 50% of admitted patients are malnourished or at high risk for malnutrition. Acutely ill patients frequently suffer from inflammation and subsequent anorexia, which leads to inadequate food intake and, therefore, to a catabolic state. Under these circumstances, the nutritional status further deteriorates, which may cause rapid weight loss [6].

The association between malnutrition and adverse clinical outcome is well described in the literature [7,8,9,10,11,12,13,14,15,16,17,18,19,20,21,22,23,24,25,26,27,28,29,30,31,32]. Nutritional treatment is urgently needed in malnourished patients to counteract negative metabolic and clinical consequences, to speed up recovery processes, and to enable better quality of life and patient autonomy [2,28,33,34,35].

**Figure 1:**
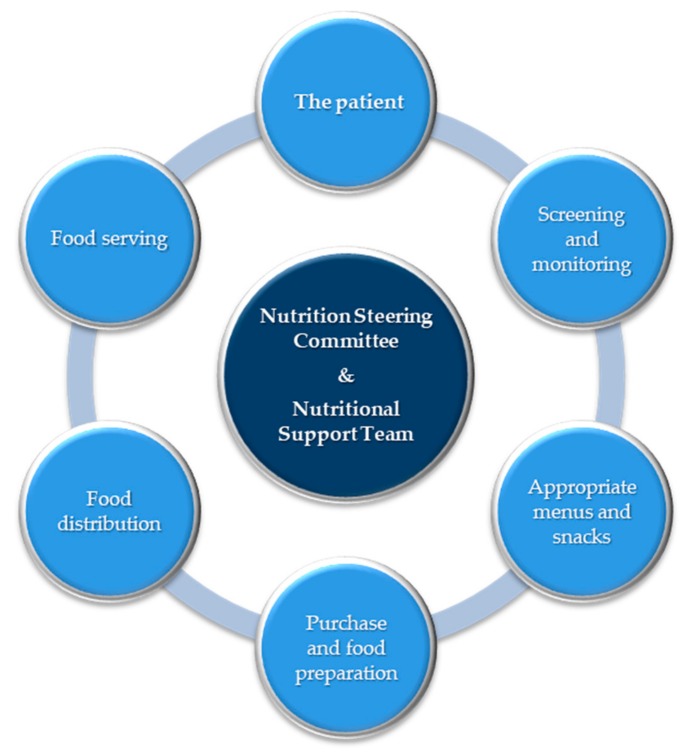
The food chain [36]. The food chain has been adopted to emphasize that all stages in the provision of food must be adequate, from screening of patients and planning of menus to the distribution and serving of the food.

The term “food chain” (Figure 1) has been adopted to emphasize that all stages in nutritional care must be adequate, from screening of patients and planning of menus to the distribution and serving of the food [36,37]. Because of the risks, and need for nutritional support, it is desirable for hospitals to appoint a multidisciplinary and multiprofessional nutrition steering group, including the clinical nutrition team, to oversee all aspects of nutritional care, from catering to artificial nutrition [36].

Appropriate, high-quality hospital food is part of a multimodal therapy that includes a wide selection of meals, snacks between meals, and the option of fortified food. The majority of hospitals in industrialized countries should be able to provide such meals, which enable patients to meet their nutritional needs. The problem is that the number of hospitals have now outsourced the hospital kitchen, e.g., to catering companies, which may make it difficult to offer best quality food to patients. Meals may, for example, be frozen and unfrozen or heated twice, which causes an important loss of quality, e.g., regarding micronutrients. Remarkably, more than 40% of meals are left on the patient’s plate and wasted, which means a patients’ food consumption meets less than 80% of their nutritional needs, and causes additional costs for the hospital [38,39].

The high prevalence of malnutrition implies a close monitoring of food intake, on the one hand, by means of adequate meal-ordering systems and, on the other hand, by sensitizing hospital medical staff to nutritional issues. The keys to better manage nutritional support in hospitals are: (1) enhanced awareness and (2) profound knowledge of this complex matter. Attention to the organization is needed from the medical staff on the ward such as to prevent interruption of meals due to procedures or rounds, and to provide support for disabled patients who need assistance with eating. Such essential tasks have been shown to improve clinical outcomes and reduce healthcare costs in several studies [40,41,42].

**Figure 2:**
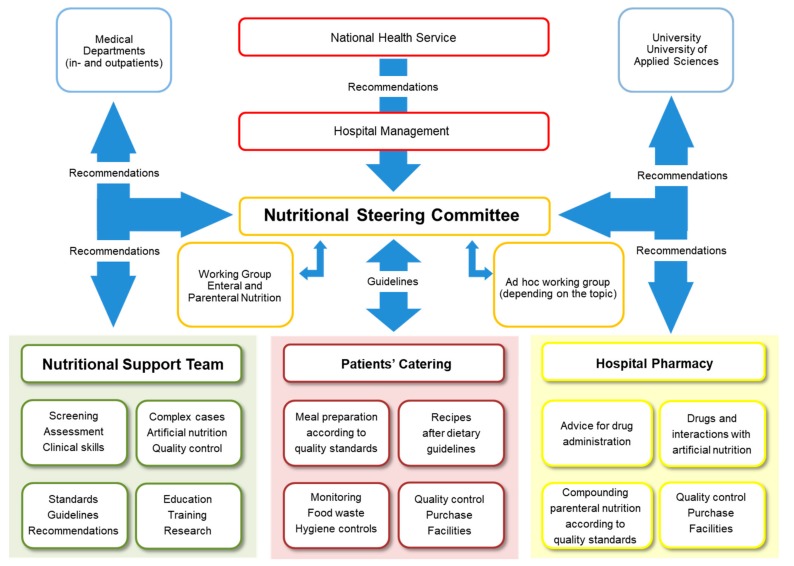
Nutritional management structure, with the Bern University Hospital, as an example.

**Figure 3:**
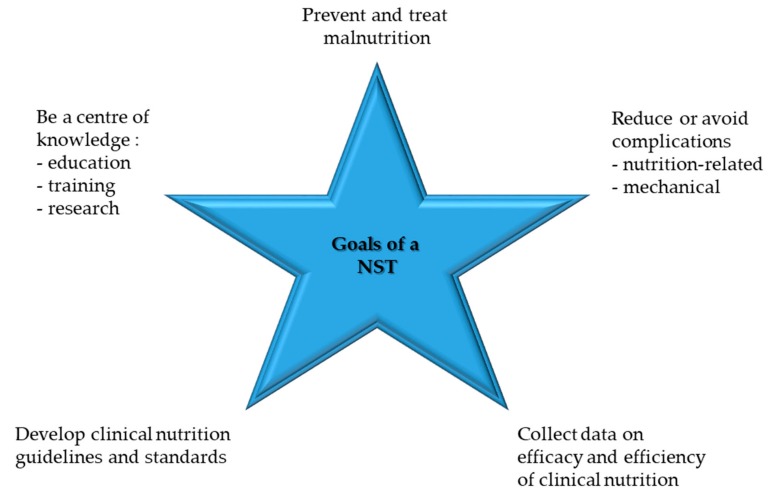
Goals of an NST, modified after [43].

In hospitals, competent nutritional management should rely on two structures: nutritional steering committees and multiprofessional nutritional support teams (NSTs) (Figure 2). The nutrition steering committee is the legislative body with direct access to hospital management (staff function). This committee consists of representative nurses, physicians, pharmacists, dieticians, cooks, managers, controllers, NST members, etc. [43]. It is responsible for promoting good nutrition as a policy, with explicit written nutritional standards, protocols, and guidelines. Further responsibilities are meant to guarantee choice of a wide range of meals and to support continuous improvement and monitoring of the nutritional therapy in terms of quality, safety, and medical efficacy [44]. This committee is also responsible for education, teaching, training, and research coordination. The nutrition steering committee and the NST should also collaborate closely with other hospitals and, in case of tertiary urban hospitals with the University as well as national and international nutritional societies, for clinical research and teaching purposes. NST exercises an executive function throughout the hospital (Figure 3). An optimal functioning institutionalized NST as described above is possible in an urban setting due to the high personal and financial resources allocated. In suburban or rural regions, it is also possible to build an NST but in a reduced format. Our long-lasting clinical experience shows that a single dietician with a physician with special interest in clinical nutrition can overtake the most important clinical tasks of an NST. There is a great opportunity to perform high standing qualitative nutritional care in any setting even if a dietician is available only once a week.

Even though there has been considerable interest in NST to improve nutritional care and, thereby, patient outcomes, there is still a lack of strong scientific evidence mainly due to only a few randomized controlled studies with small heterogeneous study populations, different treatments, and non-standardized outcomes. The aim of this narrative review is to provide an overview about composition, tasks, and challenges of nutritional support teams and discuss the current evidence regarding their efficiency and efficacy in terms of clinical outcome and cost effectiveness.

## 2. Development of NSTs

While tube feeding (enteral nutrition) has been used since the Renaissance, parenteral nutrition was first successfully used in 1968 [45,46]. At that point, nutritional therapy was established in the clinical setting. Years later, case reports on negative outcomes caused by inadequate nutritional therapies were published. Several studies showed that medical staff often overlooks the clinical signs of malnutrition, which leads to longer hospital stays and higher mortality rates [47].

NSTs were developed to counteract these concerns. At the same time, international societies such as the American Society for Clinical Nutrition (ASPEN, 1976) and the European Society for Clinical Nutrition and Metabolism (ESPEN, 1979) were created. Their primary objective was to study metabolic problems associated with acute diseases and their nutritional implications, and to provide each patient with timely and appropriate nutritional care. A study in 2005 found that NST were present in 2.8% of the hospitals in Germany, 7.9% in Austria, and 2.4% in Switzerland [48]. Ten years later, a Swiss survey indicated that 62% of the country’s hospitals had an NST composed of at least one physician and one dietician [49]. Currently, data from the “Nutrition Day Worldwide” shows that most hospitals (mostly urban hospitals) in Europe (approximately 66%) and in the USA (approximately 60%) have such NSTs. This shows the rising importance of clinical nutrition in the industrialized countries [50].

## 3. NST Composition and Collaboration 

Simon Allison (Nottingham, UK) defines an NST as a multiprofessional team including professionals from different disciplines who are good communicators and possess knowledge of the optimal delivery of nutritional therapy [51,52]. An NST improves and ensures the therapy quality and reduces health care costs by preventing needless interventions (e.g., ensuring the appropriateness of indication, stopping unnecessary long fluid therapies, preventing unnecessary catheter removals) and optimizing current treatments (e.g., combining nutritional and drug therapies) [7,23,29]. Smooth multiprofessional and interdisciplinary cooperation as well as impeccable communication are key for the success of an NST. Such a team is traditionally composed of physicians, dieticians, and nurses specialized in clinical nutrition, and pharmacists, with the dieticians primarily assuming the lead in coordinating nutritional care during a hospital stay and, thereafter, in the outpatient clinic [17,53,54,55,56].

The composition of the team may vary according to the local needs and options in terms of human resources. Additionally, intensive collaboration with hospital departments responsible for infectious diseases and hospital hygiene is advisable, particularly in the area of parenteral nutrition. This cooperation allows the NST to share knowledge and competence in catheter handling, which is important for preventing, diagnosing, and possibly treating catheter-related bloodstream infections [17]. Other specialists may also collaborate with the NST, such as physiotherapists, occupational therapists, psychotherapists, or social workers [54,56]. Multiprofessional collaboration has to be perceived as an opportunity to integrate the personal and professional expertise of each individual.

## 4. Tasks and Challenges of NSTs

**Figure 4:**
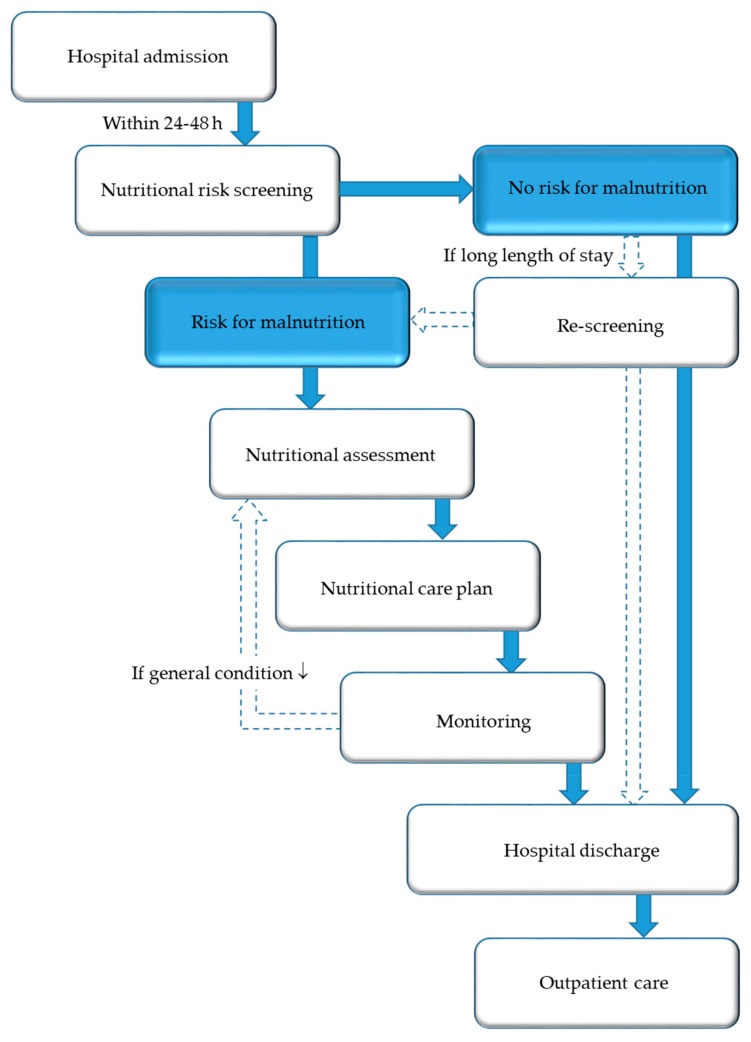
Nutritional care algorithm.

While the nutritional steering committee has a legislative role, an NST has an executive function throughout the hospital in terms of implementing standards, protocols, and guidelines in daily clinical practice. The core task of an NST is to ensure and promote high-level, evidence-based management of nutrition and to transfer this theoretical knowledge into clinical practice. The foundation of any nutritional care plan (Figure 4) is assessment of nutritional risk and early adequate provision of nutritional support to patients at risk of or suffering from malnutrition. Nutritional support is considered an essential part of the multimodal medical therapy concept, which has demonstrated good therapeutic outcomes. The individual tasks of the members of an NST are shown in Table 1.

### 4.1. Screening and Assessment

One of the most important missions of a, NST is to educate, to teach, and to train professionals in the skills related to clinical nutrition [51,53,56]. Sharing knowledge and experience with medical staff, health care providers, and students is part of effective nutritional management [55]. Awareness for malnutrition (sensitization), education, and training are, however, lacking [57]. It is a core task of the NST to implement nutritional standards, protocols, and guidelines in daily clinical practice, by establishing proper protocols for screening, assessment, and action [53,58]. The consequences of malnutrition are frequently underestimated and malnutrition is, unfortunately, rarely documented as a distinct diagnosis in medical reports and nurses’ charts despite being of central medical and economic importance in hospitals [57].

**Figure 5:**
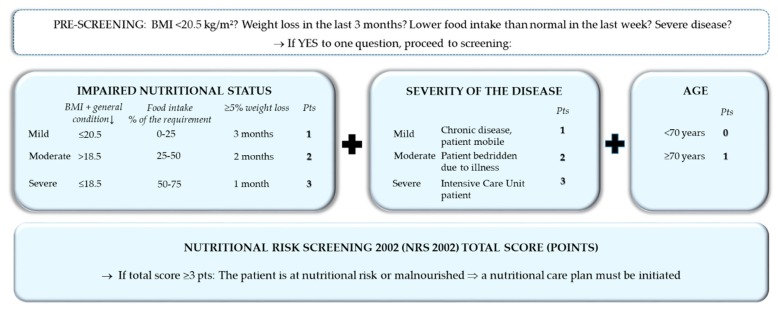
Nutritional risk screening 2002 [61].

The first step of nutritional care is the identification of patients at nutritional risk using simple, quick, and validated screening tools. Nutritional screening should be performed in all inpatients (and, preferably, also outpatients) within 24–48 h after hospital admission, respectively, as well as admission on the ward/intermediate care unit/intensive care unit. Nutritional screening should be performed by trained medical staff, at best, multi-professionally from nurses and physicians in charge of the patients, but, alternatively, from nurses, dietitians, or physicians only [59,60]. The Nutritional Risk Screening 2002 (NRS 2002) (Figure 5) is a widely used and well-validated screening tool used in hospitals to determine whether the patient is at nutritional risk [61]. If confirmed, a care plan has to be developed, based on more detailed nutritional assessment to determine the degree/severity of malnutrition. Patients with special metabolic, functional, or clinical problems that cannot be cared for by standard means should be referred to nutrition experts for more detailed nutritional assessment and design of a care plan.

Formal quantification of food intake may be helpful. Nutritional assessment can be carried out for at least two days using food diaries or food intake charts (e.g., semi-quantitative plate diagram) that are kept by nursing staff [62]. These can be used by dietitians to calculate energy and protein intake. Anthropometric measurements such as body weight, height, body mass index (BMI = weight ÷ (height in meters)^2^), and, if applicable, mid-upper arm circumference or triceps skin fold tests may also be included [62,63]. In addition, according to the local circumstances and standards of care, the following additional measurements may complete the nutritional assessment: body composition (bioelectrical impedance analysis), muscle function (handgrip strength), activities of daily living (Barthel index), quality of life (mainly questionnaires such as SF-36 [64]), and calculation of energy requirements (e.g., Harris and Benedict formula, indirect calorimetry) [63]. Routine laboratory parameters (e.g., complete blood count, lipid profile, electrolytes, and liver parameters) may give information on the patient’s nutritional state (proof of nutrient deficiency, information about the etiology of malnutrition, and follow-up of nutritional therapy), the disease severity and activity, and body composition changes to identify patients at nutritional risk [65]. However, none of these markers is very specific for nutritional deficiencies, and their medical interpretation is only possible in the context of the patient’s clinical status and history. Thus, nutrition-related factors are hardly ever the sole cause of conspicuous laboratory findings, and there is no such thing as an optimal surrogate marker for malnutrition [65].

Laboratory values must, therefore, always be interpreted in a clinical context [66]. Nitrogen balance, albumin, prealbumin, transferrin, retinol binding protein, insulin growth factor-1, creatinine-height index, and total lymphocyte count are among the few parameters that may be used to quantify malnutrition in malnourished patients [65]. Concerning micronutrients, several potential deficiencies have been associated with malnutrition, including vitamins (i.e., vitamin B12, folic acid, fat-soluble vitamins A, D, E, K) and trace elements (especially zinc, iron, and selenium). The goal of nutritional assessment is to gain an understanding of the patient’s nutritional status in order to develop a nutritional care plan containing an accurate calculation of the individual energy and protein requirements and choice of the most appropriate form of feeding (normal food, special consistency, fortified meals, snacks, oral nutritional supplements, or artificial nutrition support).

To call attention to the daily work of an NST (screening, assessment, and nutritional therapy) and, for the reimbursement of this procedure by insurance companies, it is crucial to record, document, and use the code for malnutrition in the Diagnosis Related Groups (DRG) tariff system. It is, therefore, important that the additional revenues arising from use of the DRG code for malnutrition and its therapy are reinvested to cover costs and promote NSTs.

### 4.2. Nutritional Therapy

**Figure 6:**
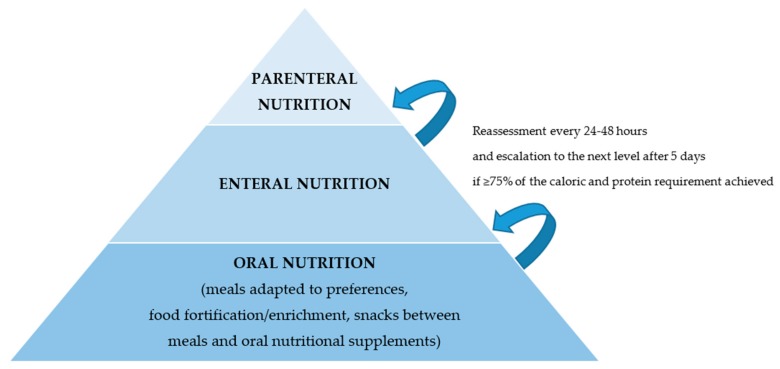
Strategy for nutritional therapy. The nutritional therapy is designed by an NST based on the patient’s needs and current situation. The most physiologic route of nutrition delivery is preferable. Nutritional therapy should be regularly re-evaluated and escalated if needed.

NST offers a hospital-wide service with the aim to improve the quality of nutritional therapies targeting complex multimorbid patients, and, in general, to “fight against malnutrition.” From a clinical and therapeutic standpoint, nutritional management starts with the identification of patients at nutritional risk—a status that will subsequently guide clinical decision-making—and focuses on those patients likely to benefit from nutritional therapy [67]. Using clinically important endpoints, there is now a substantial body of evidence showing that nutrition support improves outcome when it is implemented appropriately [28]. Thus, after an assessment has been completed and the severity of malnutrition has been determined, the attending medical staff—in cooperation with the NST—sets the individual nutritional plan and the strategy to achieve these goals (Figure 6). While the objective is always to fully meet the individual energy and protein requirements, one should strive for a nutritional intake of at least 75% of those needs [3,68]. Achievement of the goals set and adherence to therapy should be re-evaluated every 24–48 h. If necessary, the nutritional intervention should be adapted. Escalation of the support strategy—i.e., from oral to enteral or from enteral to parenteral nutrition—should be considered within five days [28].

### 4.3. Monitoring and Safety

An NST ensures the correct handling of the artificial nutrition, and reviews the appropriateness of the therapies and related prescriptions [51]. The initiation of a nutritional therapy in complex clinical situations and its proper documentation are also tasks of an NST [69]. An NST ensures the quality and safety of nutritional interventions, especially artificial nutrition, which helps to reduce potential mechanical and metabolic complications (e.g., blood glucose issues and refeeding syndrome) as well as infections [70]. The NST has a consultative role for the treating medical staff in the hospital and takes over the management of the nutritional therapy in outpatients.

### 4.4. Outpatient Management

**Figure 7:**
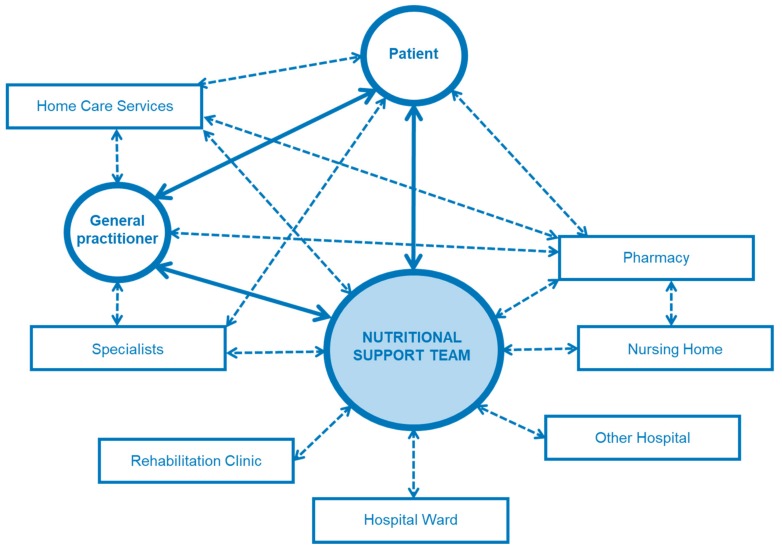
Network of an NST. NST has a central role in the management of patients with complex nutritional therapy in the inpatient and outpatient setting, during the hospital stay and beyond.

Nutritional therapy is normally initialized during the hospital stay, and continued after discharge under close monitoring. The NST plays a key role in management of the therapy during the transition from the inpatient to the outpatient setting (Figure 7). The NST carries out regular visits on the wards, and, subsequently, plans and organizes the hospital discharge from a medical as well as a therapeutic point of view. It monitors the patients, when possible, in regular consultations in the outpatient’ clinic. If artificial nutrition is needed at home, the multiprofessional NST instructs and educates patients as well as relatives and caregivers in close cooperation with the treating medical staff. After discharge, the NST remains the core contact for patients, their relatives, their general practitioners, and home care services regarding problems with nutritional therapy (intricate nutrition-related questions, complications, problems with devices, etc.). The NST also plays a central role in the outpatient setting, embedded in the complex interdisciplinary and multiprofessional therapeutic-medical network.

### 4.5. Standards and Processes

An NST monitors the clinical outcomes of the patients and regulates/optimizes processes of the nutritional intervention accordingly. Furthermore, NST periodically checks operating procedures as well as patient procedures, and proposes changes to optimize nutritional care. A good example is the transition from parenteral to enteral nutrition, or from enteral to oral therapy [58]. Since this is the platform of evidence-based practice, operational tasks of an NST include guaranteeing a wide range of meal choices, applying standards of care, implementing medical guidelines, developing standards for consultations, implementing evidence-based nutritional treatment, and maintaining high standards for the quality of hospital food (recipe management) in close collaboration with the catering department [36].

### 4.6. Education, Training, and Research

An NST also oversees and coordinates education and training in the field of nutritional management, according to the local possibilities and settings. This includes the dissemination of experience, expertise, and skills to trainees, students, and residents as well as other medical and para-medical staff. Multiprofessional work, connected thinking, and effective interdisciplinary communication are mandatory [54,55,56,71,72]. NST can ensure optimal treatment quality only when all professions and disciplines cooperate smoothly and the patient is given a place. 

A respectful and strength-based team culture is the goal. Nutritional interventions and counseling should be scientifically-based whenever possible, and should correspond to the latest knowledge (evidence-based practice). The level of evidence that informs the daily clinical practice of an NST is not always satisfactory and is often based on long-term experience and expertise. 

Beyond teaching, knowledge transfer, and skill development, NST should be able to perform clinical translational research and run clinical studies. A trial hypothesis may be generated in response to the concerns and challenges of everyday practice. Exchange of ideas, networking, and cooperation with other hospitals, universities, institutes, and societies is essential in this context, depending on the hospital setting [56].

## 5. Efficacy, Efficiency, and Positive Outcomes

An NST is often involved in defining the indications for and implementing artificial nutrition [73]. Through the involvement of an NST, there are significantly more correct indications for parenteral nutrition, and, as a result, many labor-intensive interventions can be avoided [53]. In the study of Sriram et al., the number of indicated parenteral therapies increased from 71.3% to 83.4% between 2003 and 2006 due to the intervention of NST [29]. At the same time, non-indicated interventions decreased from 16.5% to 8.9%, which is a sign of higher treatment quality [29]. Boitano et al. investigated compliance with the ASPEN guidelines for parenteral nutrition, which were implemented between 2007 and 2010 [7]. Through changes in the prescription forms, implementation of NST visits on the wards, and the education of physicians, the number of non-indicated therapies could be reduced. The percentage of indicated parenteral therapies increased from 60% to 97%, and around 85% of the patients were able to meet their energy and protein needs, versus 54% before [7]. The close monitoring of nutritional therapy showed an increase in correctly documented laboratory values from 53% to 83%. Additionally, the percentage of patients with hyperglycemia, which is the most frequent complication of parenteral nutrition, could be reduced from 47% to 3% [7]. Besides the obvious increase in treatment quality, the hospital was able to save a total of $5.3 million USD. In the study of Trujillo et al., including consecutive patients treated with parenteral nutrition, 15% of the nutritional interventions were non-indicated and 23% could have been avoided, for a total cost savings of $183,309 per year [32]. Through the interventions of an NST, metabolic complications could also be significantly reduced, from 66% to 34%, which represents $510,746 USD yearly. During 1997, in a Swiss university hospital, 69% of the parenteral nutrition prescriptions were done without involvement of an NST, and 28% of these were non-indicated [23]. Of these non-indicated therapies, 58% were inadequate. In 62% of the patients, energy intake was too low. In 20% of the patients, it was too high, and an additional 17% of patients received no vitamins or trace elements. After an NST was involved, the percentage of patients receiving parenteral nutrition decreased to 35% (2765 bags in 1995 vs 1812 bags in 1998), which leads to more enteral tube feeding. Furthermore, the number of catheter-related infections decreased from 25 (1995) to 3 (1998). Through the direct involvement of an NST, a total of 245,000 Euros per year could be saved [23]. In England, savings of over 50,715 British Pounds were achieved yearly, through the NST monitoring among medical and surgical patients [20]. Through the involvement of an NST, Chris Anderson et al. demonstrated a reduction of the yearly parenteral nutrition costs from $2107 to $1784 USD (mean total per day on parenteral nutrition) [8]. 

Already in the 1970s and 1980s, studies showed the efficacy (drop in severe catheter-related infections) and the associated cost-effectiveness of involving an NST [9,11,13,17,18,25,26]. Later on, further studies revealed that NST optimizes nutritional therapy and decreases metabolic complications. Moreover, through its interventions, NST is able to reduce the occurrence of electrolyte imbalance, especially in patients on home parenteral nutrition [44,45,46,47,57,74]. The recent study of Park et al. showed that the early intervention of an NST in critically ill patients with gastrointestinal diseases positively influences survival [22]. A significant reduction in 90-day mortality under oral nutritional therapy was reported in the study of Deutz et al. [10]. Benefits of NST interventions (oral, enteral, or parenteral) on patients’ clinical outcomes could be demonstrated in many other randomized controlled trials over the last two decades, including improved energy and protein intake, shorter length of hospital stay, fewer complications, a lower elective rehospitalization rate, less weight loss, improved muscle function, and an improvement in quality of life [12,14,15,16,19,21,24,27,28,30,51]. In the study of Johansen et al., NST was responsible for the nutritional management only in the intervention group [19]. The primary endpoint was a composite of nutrition-related factors, which may influence the length of the hospital stay (mobilization, signs of infection, complications). Energy and protein intake of ≥75% of the requirements could be achieved in 62% of the patients in the intervention group versus 36% in the control group [19]. The hospital length of stay of patients who developed complications was significantly shorter in the intervention group [19]. Nutritional therapy may be carried out easily at home with the support of home care services, which results in substantial cost savings [31].

More recently, the efficacy and efficiency of an NST (counseling, therapy, and patient procedures, according to a protocol) were confirmed in the multicenter randomized, controlled Effect of early nutritional support on Frailty, Functional Outcomes, and Recovery of malnourished medical inpatients Trial (EFFORT) of Schuetz et al. [28]. In this study, more than 2000 polymorbid medical inpatients at nutritional risk (NRS 2002 total score ≥3) were randomly assigned to either receive a standard hospital diet versus individualized nutritional support, according to a nutritional protocol [75]. After 30 days, the positive effect of the individualized nutritional management through an NST could be shown. A total of 79% of the intervention group reached their energy and protein requirements, with 76% even within three days (high compliance rate). In the control group, 54% of the patients reached their energy requirements, and 55% reached the protein requirements. Quality of life, functional status, and clinical outcome were also significantly improved. Improved outcome can be translated into a need to treat 25 patients to prevent one adverse clinical outcome and 37 to prevent one death [28]. Notably, there was no increase in side-effects or complications, such as refeeding syndrome, associated with nutritional support [76,77]. These results show that malnutrition is a mostly modifiable risk factor and that a global strategy aimed at meeting the needs of individual patients is of decisive importance. Cachexia may not be fully reversed with nutritional support but remains essential until refractory cachexia occurs [78,79] (Table 2).

## 6. Strength of Evidence Regarding Nutritional Support Teams

Evidence-based medicine is intended to optimize the decision-making of physicians and patients by emphasizing the use of evidence from well-designed and well-conducted research including typically randomized trials and meta-analyses summarizing effects of such single trials. For many fields of clinical nutrition, including NSTs, there has been an important lack of large-scale interventional studies providing such high-quality evidence, and much of today’s knowledge is based on observational research and experience of physician and dieticians. As a consequence, current clinical practice guidelines, often give weak recommendations regarding nutritional topics. However, as outlined above, there are some new and important clinical trials in the field, which provide strong evidence in favor of nutritional support and thus also in favor of NSTs [10,28]. However, there is clearly room for further improvements in our understanding on how to best use nutrition in individual patients. 

## 7. Conclusions and Outlook

Malnutrition is a mostly modifiable condition with potentially deleterious consequences, if left untreated. Malnourished patients can be detected early and treated in a timely fashion through comprehensive nutritional care management. This contributes to improvements in the patient’s clinical outcome, as recently shown in the EFFORT trial [28]. An interdisciplinary approach and nutritional therapies are effective in cost containment (improving quality of treatment, avoiding unnecessary interventions, and simplifying management), which is especially relevant for the modern healthcare policy. These results show that NSTs should be widely propagated and implemented in a hospital. There is growing evidence from clinical trials demonstrating the efficacy and efficiency of NSTs. The success of nutritional medicine strongly depends on their institutionalization and visibility of the field and the role of the NSTs in modern multimodal medical care. The key task of NSTs is to implement a comprehensive nutritional care system, so that every patient who could potentially benefit from nutritional support receives it rapidly, adequately, and with the highest standards of quality.

## Figures and Tables

**Table 1:** Individual tasks of NST members [43].

Professional Function	Nutritional Care Tasks
Nurse	Gives advice on routes, methods, and systems for delivery of enteral/parenteral nutrition; Assesses adequacy of access to nutrition therapy; Advises on use of tubes, feeding pumps, and enteral/parenteral equipment; Implements and adapts protocols on methods of delivering enteral/parenteral nutrition to establish uniformity, save costs, and prevent mechanical complications; Educates on enteral/parenteral nutrition and highly complex nutritional therapy; Conducts research on complex nutritional therapy.
Physician	Prescribes and manages enteral/parenteral therapy; Promotes the established nutrition therapy within the host specialty; Provides professional input for highly complex nutritional therapy; Supports ongoing research and projects on complex nutritional therapy; Informs colleagues, physicians in training, and members on the board of directors of the importance of good nutrition therapy on the ward.
Dietician	Gives advice on enteral/parenteral nutrition (indications, choice of feeding solution, nutritional goals); Advises about options for enteral/parenteral nutrition and other nutrients (e.g., immuno-nutrition, vitamins, trace elements); Edits, implements, and adapts protocols on enteral/parenteral nutrition and complex nutritional therapy; Develops and interprets screening tools; initiates and performs nutritional assessment; Contributes to education and conducts research on complex nutritional solutions.
Pharmacist	Provides logistical support for parenteral nutrition; Oversees and provides information about possible chemical/pharmaceutical interactions between parenteral nutrition components; Provides professional input on the composition of parenteral nutrition, on stability and compatibility of parenteral admixtures, and on drug/medication interactions with enteral/parenteral nutrition; Supports ongoing research projects on complex nutritional therapy, develops and implements parenteral nutrition protocols.

**Table 2:** Clinical studies showing efficacy and efficiency of NST.

Outcome	Correct Indication	Cost Savings	Decreased Complication Rates	Reduced Mortality	Improved Monitoring	Increased Dietary Intake	Positive Influence of Individualized Nutritional Support
**Studies**	Boitano et al. [7] Piquet et al. [23] Trujilo et al. [32] Sriram et al. [29]	Boitano et al. [7] ChrisAnderson et al. [8] Curry et al. [9] Faubion et al. [11] Goldmann et al. [13] Jacobs et al. [18] Kennedy et al. [20] Piquet et al. [23] Ryan et al. [25] Sanders et al. [26] Trujilo et al. [32]	Boitano et al. [7] Curry et al. [9] Faubion et al. [11] Gariballa et al. [12] Goldmann et al. [13] Ha et al. [14] Hegerova et al. [15] Hickson et al. [16] Jacobs et al. [18] Johansen et al. [19] Norman et al. [21,41] Piquet et al. [23] Ruefenacht et al. [24] Ryan et al. [25] Sanders et al. [26] Somanchi et al. [27] Schuetz et al. [28] Starke et al. [30] Stratton et al. [31] Trujilo et al. [32] ten Dam et al. [43] Dudrick et al. [45] Fürst et al. [46] Butterworth [47] Allison [51] Council of Europe [57]	Park et al. [22] Schuetz et al. [28] Deutz et al. [10]	Boitano et al. [7] Kennedy et al. [20]	Boitano et al. [7] Gariballa et al. [12] Ha et al. [14] Hegerova et al. [15] Hickson et al. [16] Johansen et al. [19] Norman et al. [21,41] Ruefenacht et al. [24] Somanchi et al. [27] Schuetz et al. [28] Starke et al. [30] Stratton et al. [31] ten Dam et al. [43] Dudrick et al. [45] Fürst et al. [46] Allison [51] Council of Europe [57]	Johansen et al. [19] Ruefenacht et al. [24] Schuetz et al. [28]
